# Progress in Surgery

**Published:** 1895-07-13

**Authors:** 


### Progress in Surgery.

### CEREBRAL SURGERY.

(*Continued from page* 239.)

The mortality of operations for intracranial hæmorrhage has considerably lessened since 1886. When Jacobson wrote his paper the mortality in cases of extradural hæmorrhage was 81 per cent., but in the cases collected by the author it is only 30 per cent. Some very interesting and remarkable cases are given. In one there was no loss of consciousness after the accident (a fall of eight feet from a ladder), and no head injury was suspected, the patient continuing his business. On the eighth day he became comatose, with twitching movements of the face and both sides of the body. The left pupil was dilated and fixed. Six ounces of extradural clot were removed from the region of the left middle meningeal artery, and next day he became conscious. Another case quoted can hardly be said to be one of hæmorrhage, but it is very interesting. A boy received a blow over the right ear with a cricket ball at five p.m., but was only a little giddy, and continued to play. At eight p.m. he was comatose, and three days later paralysis began in the left leg, and spread to the left upper extremity and side of the face. He was trephined over the motor area, and a fissure of the internal table discovered, but hardly any hæmorrhage. The removal of the bone relieved pressure, and he gradually recovered. In the case of a pistol wound of the left temporal region the only symptom was aphasia, and this was due to extradural clot from damage to the middle meningeal artery from fracture of the inner table. Complete recovery followed its removal. In looking through the group of cases of subdural hæmorrhage one is struck by the length of time which often elapses between the injury and the onset of symptoms—three days to a week frequently, and in one case on the twelfth day. In these cases much of the blood remains fluid. The clot, or collection of fluid blood, was found generally by indications of its pressure on the motor area. Dr. Walton[8] mentions a case of ruptured middle meningeal artery in which five weeks of apparent health intervened between the infliction of the injury and the onset of the coma, and the same writer states that the longest interval on record between the time of receiving the injury alleged to be the cause of the hæmorrhage and the onset of symptoms is three months. He points out that in these cases paralysis may occur without convulsions preceding, and that bilateral rigidity does not necessarily point to hæmorrhage over both hemispheres, but may result from extensive unilateral middle meningeal hæmorrhage, but in such a condition the onset will be unilateral. The prognosis of aphasia or hemiplegia from cortical hæmorrhage he considers good, even if there be considerable laceration, if the blood is evacuated. He thinks diagnosis of the side of the hæmorrhage occurring from rupture of the middle meningeal artery may be made from paralysis of the third nerve alone, if general symptoms of hæmorrhage are present. Dr. Putman[9] records a case of hæmorrhage under the pia mater over the motor area, following a fall on the head, which gave rise to jerking movements on the opposite side, followed by paralysis and the gradual onset of coma. The symptoms came on twenty-four hours after the fall, and the patient died nine days later. Trephining over the motor area was evidently indicated. Dr. Putman remarks that the absence of any showing of the pulse was to be noted in this case, and he considers that this is often so in cases of compression. "Trephining for Middle Meningeal Hæmorrhage without Fracture of the Skull" is the subject of a paper by Dr. W. J. Taylor, of Philadelphia,[10] in which he states that it is almost impossible to say whether the clot is extradural or subdural in the same area by the localising symptoms alone, and advises that an opening should always be made in the dura mater to determine this point, provided the clot is not found directly beneath the bone, as such an exploration does not increase the dangers of an aseptic operation. A very interesting case is recorded in which there were symptoms on both sides—convulsive movements on the right and paralysis of the left angle of the mouth, and paresis of the left side. Dr. Taylor first removed a disc of bone from over the right motor area, but there was no hæmorrhage there, though on opening the dura mater an ounce of cerebro-spinal fluid escaped. On the left side the middle meningeal artery had ruptured, and there had been much extradural hæmorrhage. Although the bleeding artery was tied and the clot removed, the patient died in 48 hours, though the autopsy showed no other injury of the brain. A case of cortical hæmorrhage causing hemiopia is also reported.[11] It was associated with a fall on the head, and Dr. Keen, who saw the case, was inclined to think there was extradura clot from rupture of the posterior branch of the middle meningeal artery, but the post-mortem examination made it probable that the woman fell as a consequence of the hæmorrhage.

Dr. Phelps contributes a series of papers on the differential diagnosis of traumatic intra-cranial lesions,[12] based on the collected records of 176 cases. It is a sequel to a paper based on a study of 124 cases, read before the New York State Medical Association two years before. He considers that elevation of temperature is the unfailing manifestation of traumatic lesions within the cranium. Where the temperature is easily lowered he thinks shock is present, and would base the differential diagnosis between head injury and alcoholic coma on the elevation of temperature in the one and its depression in the other. Of a large number of cases of fractured base of which Phelps has collected the records, external hæmorrhage was noted in four out of five. He does not think that the difficulty of distinguishing hæmorrhage under the conjunctiva, from the nose or ear, from local injury or fractured base, is very great. Intra-cranial hæmorrhage after injury the author does not consider is often an isolated lesion, but he thinks it is of frequent occurrence in all forms of injury, and in nearly 60 per cent. of his collected cases it has been an important factor in the causation of symptoms, and in one-third of this percentage it has been the direct cause of death. The two symptoms which have been considered of great importance in the diagnosis of intra-cranial hæmorrhage—loss of consciousness after some appreciable interval and dilatation of the pupil, Phelps does not think his collected cases show to be really of any great value. The change in the pupils has varied much in character, and cannot, he thinks, be regarded as typical in any of its forms. The post-mortem examinations in these 176 cases seem to show that lesions of the frontal lobes produce impairment of the intellectual faculty. The special manifestations of disordered intellect which they presented were loss of memory, especially of the manner of the injury, confusion of ideas, inability of comprehension, incapacity of mental concentration, incoherence, delusions, apathy, or stupor. The lesions were usually of the nature of laceration. In very few cases indeed was the injury other than in the frontal lobes when such impairment of mind was present. Lesions of the tempero-sphenoidal lobe, except in one instance, have not given any localising indication of their presence. Aphasia (the form is not mentioned)

was present once in a cortical laceration of the first and second left temporal convolutions. Phelps does not consider hæmorrhage pressing on the centres of speech is ever a cause of aphasia, though cases have been published in support of such a view. In such cases he thinks there has always been a laceration, possibly overlooked when the hæmorrhage was discovered. He points out that the removal of a small portion of bone not infrequently relieves morbid cerebral conditions, though the lesion remains undiscovered, and refers to one case in this series in which traumatic convulsions of several days' continuance were immediately and permanently controlled by trephining, though nothing was discovered. Lacerations of the cerebellum, though numerous, afforded no distinctive symptoms. The effect of lesions of the medulla is of great interest in connection with Professor Victor Horsley's paper, previously referred to. Hæmorrhage, causing compression, caused infrequency of respiration. In three instances in which the respiratory acts were reduced to no more than two in the minute, radial pulsation was continued for two and three minutes after respiration had entirely ceased. In some cases very high temperatures were found associated with laceration of regions which corresponded to the heat centres determined by experiment, but these were always included in more extended cortical lesions, and Phelps has observed equally high temperatures, where the laceration has existed in parts unsuspected of special influence over the control or production of heat. He thinks high elevation of temperature from traumatic laceration of the brain is dependent on general nutritive changes rather than on lesions of limited thermogenetic or thermotaxic centres.

In place of hæmorrhage Phelps has sometimes found recorded the existence of a subarachnoid serous effusion, but it was associated with more serious organic changes, so that it was impossible to connect it with any symptom. Laceration of the brain, he thinks, seldom occurs without hæmorrhage, often sufficient to have material effect in the production of the symptoms. Only in ten cases was cortical hæmorrhage or general contusion insufficient to cause symptoms, so that the result of the laceration could be discovered. In these ten cases consciousness was ordinarily lost in the beginning, delirium frequently followed, the patient became irritable, and lost control of his urine and fæces, the condition of the pupils remaining variable. There was remarkable primary elevation of temperature following recovery from shock, and in early fatal cases rapid and progressive increase of temperature. The high temperature was maintained in the presence of complications. Convulsions were of frequent occurrence in the fatal cases. The author never knew of these occurrences in general contusion; and in hæmorrhage they result from compression of the motor area and are followed by paralysis, whereas in these cases of laceration they were the result of lesion of the frontal or temporo-sphenoidal lobes.

Central cerebral abscess only occurred twice in a series of three hundred cases. Both were associated with compound depressed fracture of the vertex without wound of the dura and were situated some distance from the injury. In the first case the bone was elevated directly after the accident and no symptoms developed until the twentieth day. There was slight mental dulness with facial paresis. A few drops of pus escaped from the wound and pus was found two inches in the brain. In the second case the wound had healed, when on the twenty-second day the patient suddenly began to suffer from frontal pain and became drowsy, delirious, and hemiplegic, with normal temperature. Phelps considers that surface cerebral abscess or hernia cerebri after wounds has practically disappeared from the field of American surgery.

[8] American Journal of Medical Sciences, April, 1895, p. 407. [9] Ibid, p. 494. [10] Therapeutic Gazette, Oct. 15th, 1894. [11] Ibid. [12] New York Med. Journal, 1894, Nov. 10th, p. 578; Dec. 8th, p. 710; Dec. 15th, p. 741; Dec. 22nd, p. 779; Jan. 5th, p. 8.

